# Dispersal and competitive release affect the management of native and invasive tephritid fruit flies in large and smallholder farms in Ethiopia

**DOI:** 10.1038/s41598-020-80151-1

**Published:** 2021-01-29

**Authors:** Tibebe Dejene Biasazin, Tadiwos W. Wondimu, Sebastian Larsson Herrera, Mattias Larsson, Agenor Mafra-Neto, Yitbarek W. Gessese, Teun Dekker

**Affiliations:** 1 Chemical Ecology Unit, Department of Plant Protection Biology, Swedish University of Agricultural Sciences, P.O. Box 102, 230 53 Alnarp, Sweden; 2 Department of Zoological Sciences, Addis Ababa University, P.O. Box 1176, Addis Ababa, Ethiopia; 3 ISCA Technologies, 1230 W Spring Street, Riverside, CA 92507 USA

**Keywords:** Chemical biology, Chemical ecology

## Abstract

African horticulture is seriously affected by fruit flies, both native and invasive. Novel sustainable control methods need testing against the backdrop of smallholder-dominated farming of Africa. We evaluated the potential of male-specific attractants (parapheromones) laced with insecticide to suppress the alien invasive *Bactrocera dorsalis* and native *Ceratitis capitata*. In large-scale guava, methyl-eugenol (ME)-bait stations combined with toxic protein baits suppressed *B. dorsalis* within 8 months but resulted in a resurgence of the displaced *Ceratitis capitata*. In smallholder farms, intervention using SPLAT-ME laced with spinosad was surprisingly unsuccessful. Subsequent mark-release-recapture experiments showed high dispersal rates of flies, covering many times a typical farm size, leading to a continuous influx of flies from surrounding areas. Several other factors important for intervention were evaluated. SPLAT-MAT-ME dollops remained attractive for over two weeks, although gradually becoming less attractive than fresh baits. Further, competitive displacement was observed: *C. capitata* selectively emerged from fruits in which *B. dorsalis* infestation was low. Finally, we evaluated whether ME could be combined with *C. capitata* male attractants [trimedlure (TML) and terpinyl acetate (TA)] without affecting attraction. Combining male lures did not affect catches directly, although at very high populations of *B. dorsalis* attracted to ME interfered with *C. capitata* trap entry. Although ME-based methods can effectively suppress *B. dorsalis*, they were not effective at single smallholder scale due to the high dispersive propensity of tephritids. Further, competitive release implies the need for a combination of lures and methods. These observations are important for developing control schemes tailored for African smallholder settings.

## Introduction

Organisms that share resources in an ecosystem are often in direct or indirect competition, affecting each other’s fecundity, growth and survival^1^. Arrival of an invasive exotic species could increase competition to such a degree that it may cause competitive displacement of native species^2–4^. Numerous behavioral, physiological and ecological factors determine the success of an alien species in a new geographic area^5,6^. Efficiency in resource utilization, rapid population growth and short reproduction cycles, low pressure of natural enemies, high dispersal ability, and phenotypic plasticity are among the traits frequently observed to favor an invasive alien species over indigenous guilds^7–9^.

The family Tephritidae includes several polyphagous fruit fly pests, some of which have spread outside their native range and become invasive^2,3,10,11^. Human activities and global trade of agricultural produce have played an important role in introducing alien fruit fly species into new regions^12^. Many of these regions, however, already had indigenous or other invasive tephritid fruit fly species affecting agricultural production^2^. As a result, an invasive species may create a competitive challenge in the established resident community. In the last two decades several fruit fly species of Asian origin have invaded sub-Saharan Africa threatening fruit and vegetable production^13^. Of these, the species with arguably the biggest impact in the region is the Oriental fruit fly, *Bactrocera dorsalis* (Hendel), which was incidentally detected in Kenya in 2003 during routine fruit fly surveillance^14^. Since its detection, the pest has spread rapidly throughout the continent, causing serious damage in both large commercial enterprises, as well as in small-scale fruit production areas^15–17^ and displacing native tephritids, such as *Ceratitis cosyra* (Walker) and *Ceratitis capitata* (Wiedermann)^3^.

Fruit damage due to the native tephritid fruit fly species in East Africa was already high, causing serious problems to growers. This situation was exacerbated with the invasion of *B. dorsalis*, with damage frequently reaching 100% in the absence of effective pest management^3,18,19^. This highly invasive species has since displaced local fruit fly species from smallholder crops^3^. The relative economic impact is perhaps strongest felt by smallholder farming families that count on the small-scale sales of high-value crops to significantly contribute to the family economics. Pest management and control strategies should thus be amenable to both large-scale productions and small-scale farming, with emphasis on the latter. However, little is known about the potential of adoption and utilization of fruit fly control techniques in smallholder farming settings.

Various management techniques exist that can be used in fruit fly suppression, of which orchard sanitation and toxic bait sprays (e.g. GF-120^17^), are mainstays. Of the upcoming techniques that have a good potential of adoption by the East African farming community is the use of male attractants in a form called male annihilation technique (MAT), which selectively removes males from a population and thereby reduces the percentage of fertile females and oviposition. The technique makes use of the fact that males of many tephritid fruit flies are strongly attracted to certain chemical compounds, the ecological importance and evolutionary origin of which is only partially understood^20^. These male-specific attractants, frequently referred to as parapheromones, can be used in combination with killing agents, such as spinosad, to reduce a population from a given area through male annihilation^21^. Males of *B. dorsalis* are highly attracted to methyl eugenol (ME), whereas the native *C. capitata*, as well as some other *Ceratitis* species, are attracted to trimedlure (TML) and terpinyl acetate (TA)^20^. Success of MAT in control of fruit flies has been mainly reported for those species of males that respond very strongly to male lures. ME-based MAT has been successfully used to eradicate or significantly suppress *B. dorsalis* flies^21–23^. Although Ceratitis male lures (TML & TA) are less attractive compared to ME, potential use of these male lures for *Ceratitis* spp in MAT has been reported in Africa and Hawaii^24,25^.

In this study, the overarching goal was to understand what the possibilities and constraints are of fruit fly intervention in smallholder farms compared to large farms. Factors that were assessed included competitive displacement, competitive release, the dispersive capability of flies and the longevity of the lures. Using ME-based fruit fly intervention in both large-scale and small-scale orchards, we evaluated the rate and range of dispersal of male *B. dorsalis*. Further, we assessed if selective suppression of the invasive *B. dorsalis* using ME could cause competitive release of displaced native tephritid species, through measuring capture composition of traps pre and post intervention, as well as the composition of fruit flies emerging from various fruit species. The results are important for designing intervention campaigns in smallholder and large farms.

## Results

### Selective suppression of *B. dorsalis* in large-scale farming setting leads to competitive release of *C. capitata*

**Figure 1:**
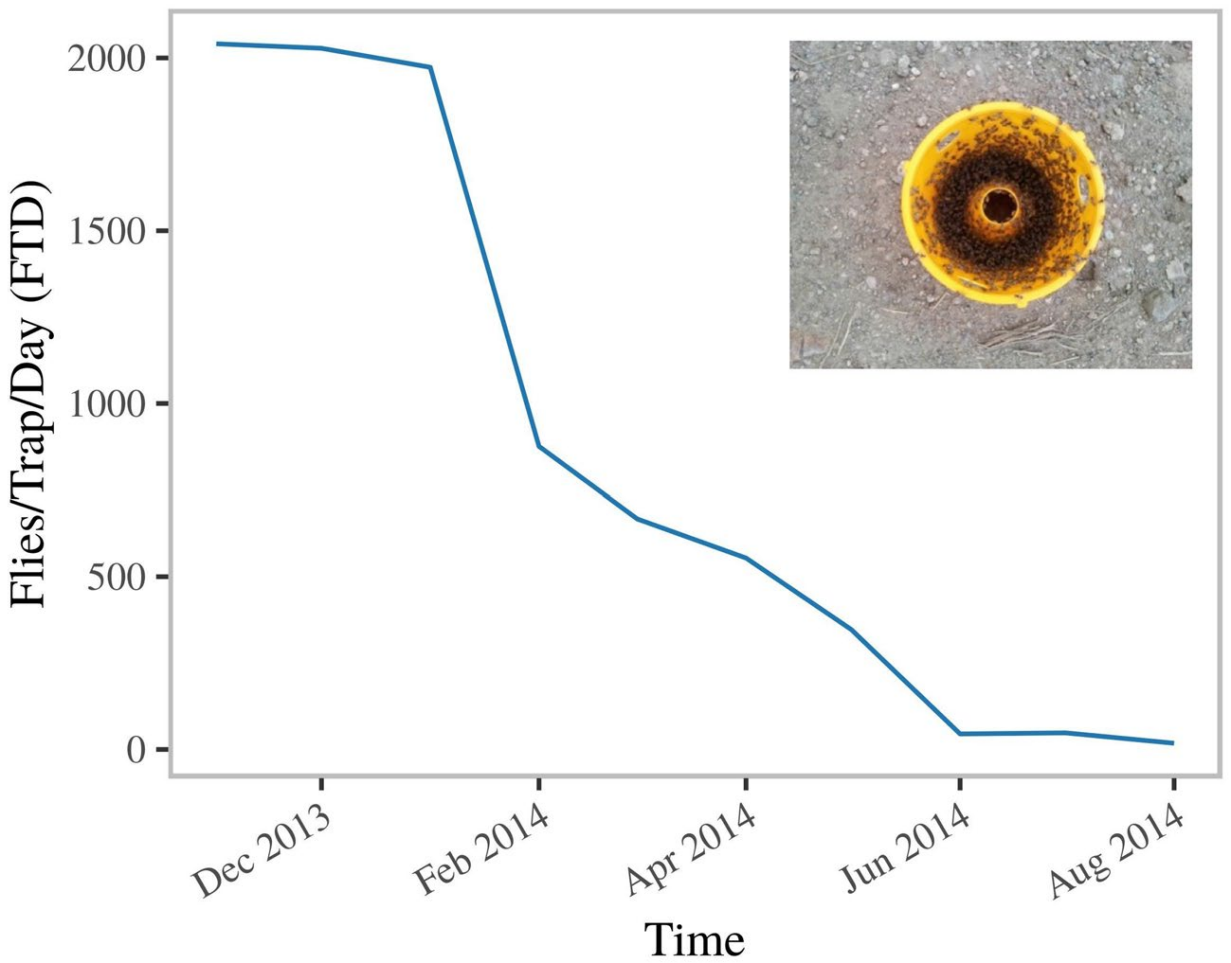
Effect of male lures, bait sprays and orchard sanitation on the *B. dorsalis* trap catches with methyl-eugenol baited traps from November 2013 to August 2014 in a large-scale farm. The inset illustrates a 30 min trap capture of a methyl eugenol trap placed 10 m outside the guava orchard prior to intervention (September 2013).

In 2013 the population of *B. dorsalis* in UAAIE was very high, to the extent that a single ME trap captured thousands of male flies in less than 30 min in an area bordering a guava orchard (Fig. 1 inset). Flies emerging from collected guava fruits consisted of 99.8% of *B. dorsalis* and only 0.002% being *C. capitata* (n = 20 guava fruits, *C*. *capitata* = 2, *B. dorsalis* = 1340). Using a combination of three management methods (M3 bait station for male annihilation, GF-120 bait sprays containing Spinosad, and orchard sanitation) the large scale farm management targeted the fruit fly population in Upper Awash, resulting in a severe suppression of the population of *B. dorsalis* within eight months (Fig. 1) and a resurgence of the population of *C. capitata* (Fig. 7A). At the start of trials in guava fields, the catches of *C. capitata* were already higher than that of *B. dorsalis* and remained so throughout the 35-week intervention period (Supporting information Fig. 1).

### ME-based intervention in smallholder farms did not suppress *B. dorsalis* populations

**Figure 2:**
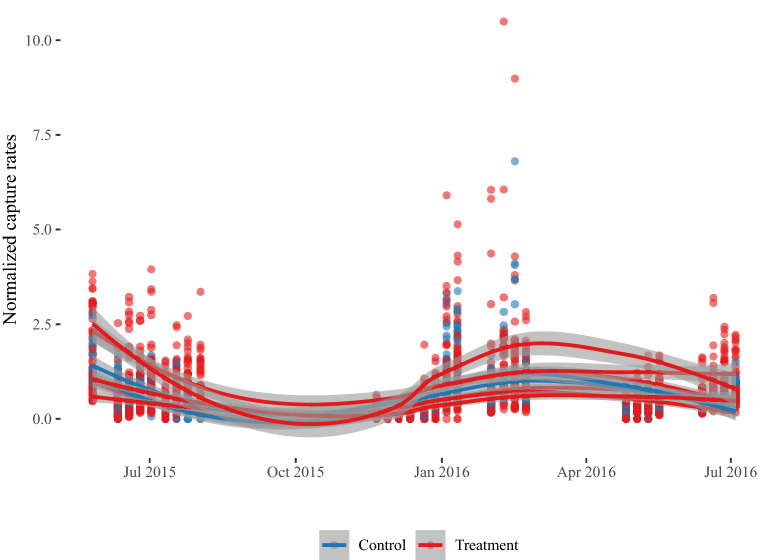
Normalized trap captures of *B. dorsalis* in Arba-Minch in control and treatment plots of smallholder orchards throughout the intervention period. Capture rates were expressed as a fraction of the average capture rates of that plot at the beginning of the intervention trial. Results are expressed as a square root transformed value. Local Polynomial Regression Fitting was used for smoothing the curves.

During 2015 and 2016 an intervention trial using ME-dollops was rolled out to assess the possibility of suppressing *B. dorsalis* in individual smallholder farms. Catches of *B. dorsalis* fluctuated considerably along with the fruiting period of mango and some other climatic factors (e.g. a flood that inundated most of the mango growing area for a few weeks). Regardless, ME-based intervention did not have a noticeable effect on population levels (measured through catches using ME-baits) compared to control plots (red versus blue lines, Fig. 2, see also supporting information figure S1). The lack of suppression could be due to the relatively small size of the fields and the diverse cropping systems, which provide for alternative hosts for *B. dorsalis*. In addition, a continuous immigration of *B. dorsalis* from neighboring fields could have underlined the lack of control. Therefore, we assessed the dispersive range of *B. dorsalis* (see below).

### *B. dorsalis* disperses over distances covering many smallholder farms

**Figure 3:**
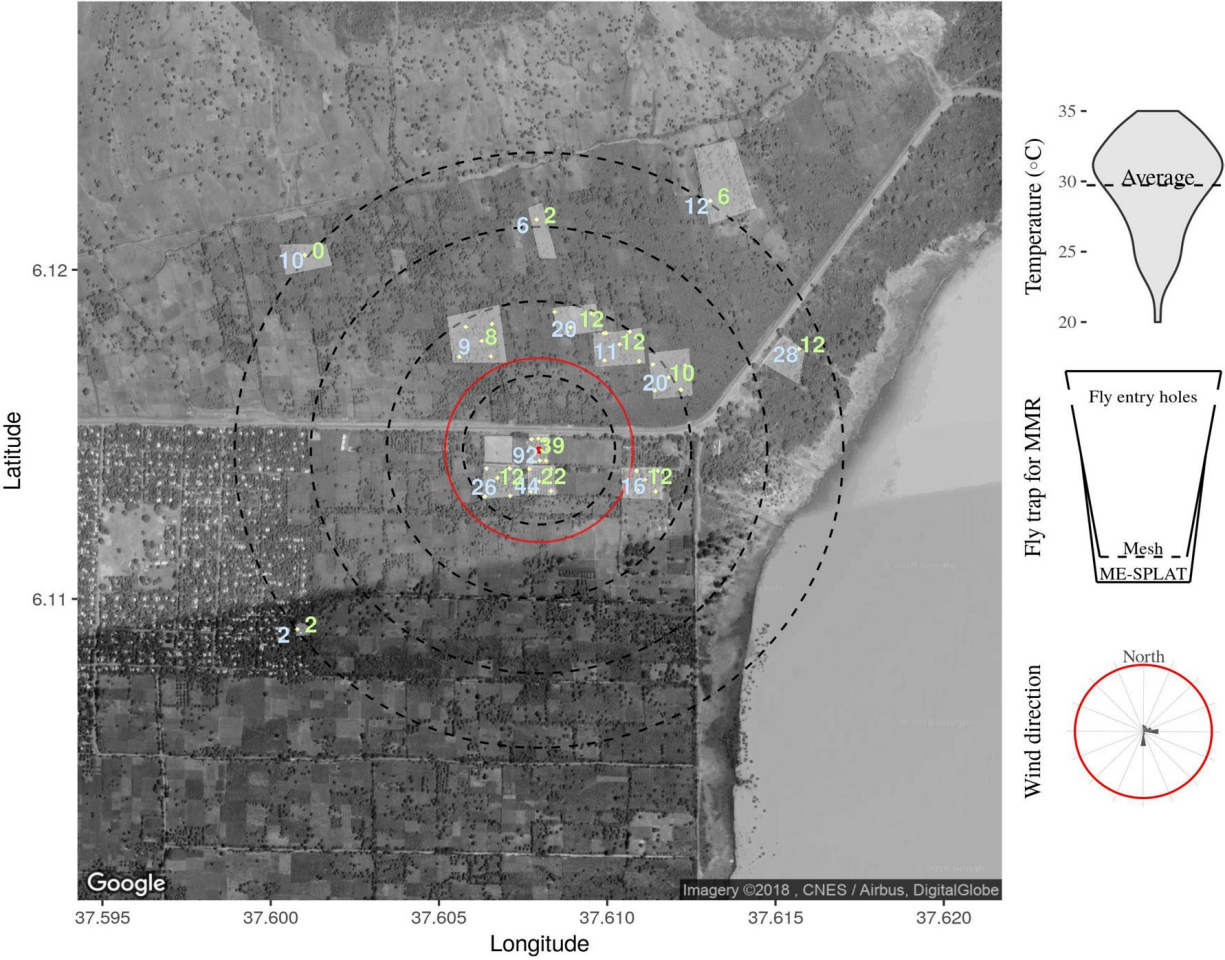
The average number of blue and green-marked male *B. dorsalis* recaptured per position. Fruit flies were either released 3 days before (green) or after trap set up (blue) from the central position (red dot). Light-shaded areas indicate the perimeters of the smallholder farms in which traps were placed. Concentric dotted circles indicate the distances from the release point (250, 500, 750 and 1000 m). For comparison, the red circle indicates the approximate size of the guava farm in a commercial farm Upper Awash. Note the lake at around 150 m east from the release point. Top inset: violin plot showing the range of temperatures at 12 noon during the observation period with an average temperature in a dashed line. Middle inset: the modified bucket trap for collecting flies for marking. Flies could not reach the methyl eugenol bait. Bottom inset: prevailing wind direction and strength (red line demarcates wind strength between calm and moderate). For visualization purposes a map was produced using the average trap catch average per location and the package ggmap, a wrapper for ggplot2 (Version:3.0.0, URL: http://ggplot2.tidyverse.org) was used for accessing the google API and downloading the map for subsequent mapping^28^, temperature and wind data was gathered using the package WeatherData^29^, where a temperature set was filtered for 12:00 h local time. The map was annotated with each of the orchards and trap position and concentric circles representing the distances from release point.

**Figure 4:**
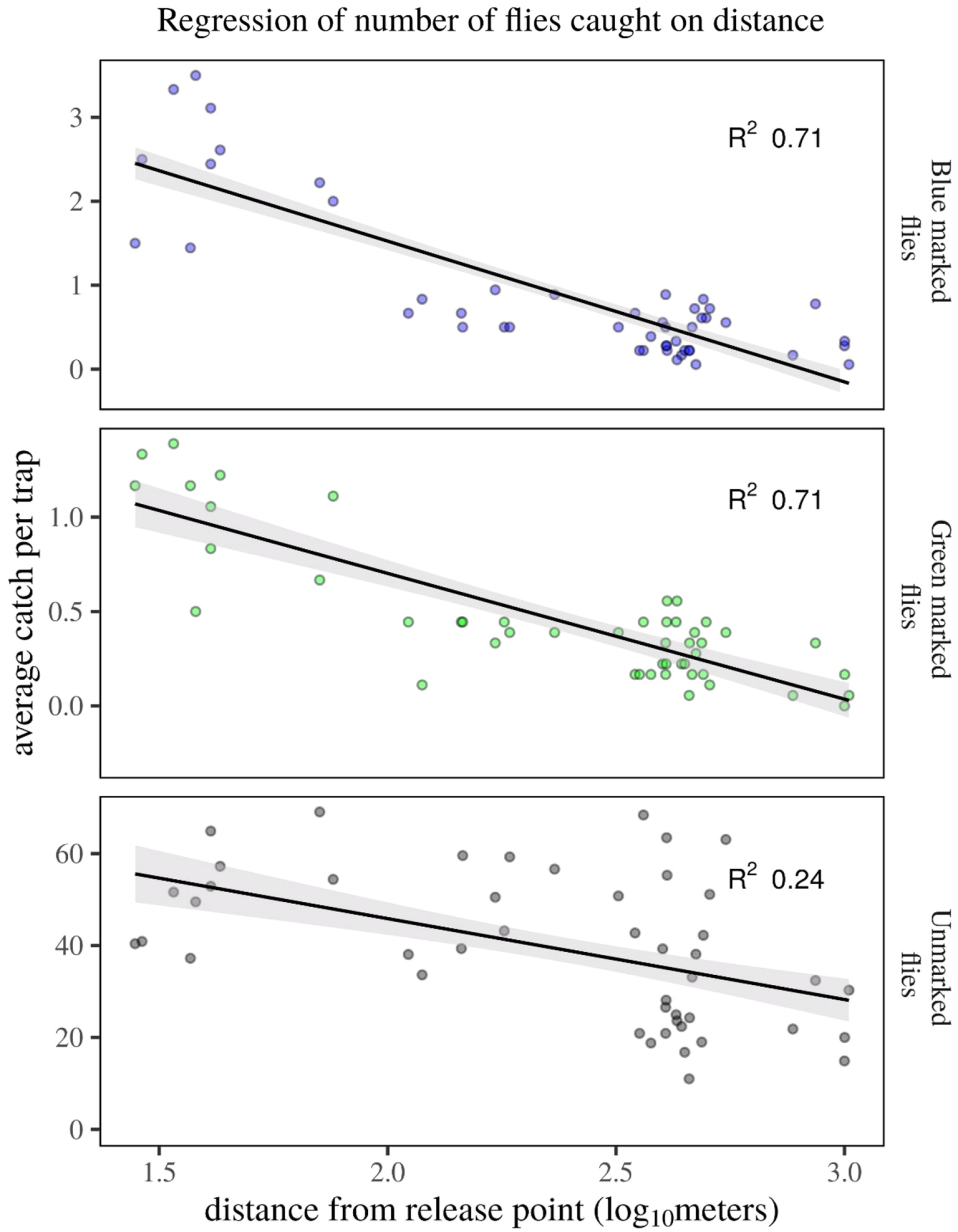
Regression of the number of recaptured *B. dorsalis* males on distance from the release point. . The number of recaptured flies declined with distance from release point (r^2^ = 0.71, F(1,46) = 112.7, p < 0.001), both when trapping started immediately after release (blue-marked flies) and when release preceded recapture by 3 days (green-marked flies). As a control, capture of unmarked flies over distance showed a weak correlation (r^2^ = 0.24, F (1,44) = 13.69, p < 0.001).

The mark-release-recapture data shows that both green (released 3 days prior to placing recapture traps) and blue-marked flies (released 24 h prior to trap deployment) were recaptured from traps at all distances and directions (Fig. 3). A total of 386 (2.96%) green and a total of 763 (6.6%) blue-marked *B. dorsalis* flies were recaptured from all releases (13,033 green-marked and 11,528 blue-marked). Of the marked flies, 11.1% ± 1.2 remained in the release bucket for longer than one hour and were excluded from the study. Flies dispersed distances many times the typical size of smallholder farms and throughout the study area, despite having ME traps at or nearby the release plot (Fig. 3). The number of recaptured flies significantly decreased with distance from the release point (Fig. 4). Whether traps were placed shortly after (blue flies) or 3 days after release (green-marked flies) dispersal distance was similar (r^2^ = 0.71, F(1,46) = 112.7, p < 0.001, and r2 = 0.71, F(1,46) = 112.4, p < 0.001, resp.), indicating placement of traps directly following release of flies did not lead to an underestimation of dispersive propensity. A weak negative correlation was found in the number of unmarked flies caught at different distances (r^2^ = 0.24, F (1,44) = 13.69, p < 0.001). Temperature was constant and the wind was ‘calm’ (less than 0.5 m/s) for most of the time (Fig. 3).

### ME formulations remained attractive for weeks

We subsequently tested the attractiveness of SPLAT-ME as a slow release formulation for methyl eugenol. Lures that had been exposed to field conditions for different lengths of time were compared at close range (1 m separation) or spaced (30 m from each other). When traps were placed in close proximity to each other, traps containing 1-day old lures (fresh lures) were as attractive as 5-day old lures, and more attractive than 7-day old lures. Lures aged for 1, 5 & 7- days were more attractive than 15-day and 30-day old lures (p < 0.0001). However, the 15-day old lure was more attractive compared to the 30-day old lure (p < 0.001).

In a separate experiment we assessed the potential longevity of SPLAT-ME by hanging traps with differently aged lures at 30 m distance from each other. Here differences between capture rates of lures of different ages were less pronounced. Thirty-day old lures and 15-day old lures were less attractive than younger lures (p < 0.0001 compared to 1, 5 & 7 day). However, 15-day old lures caught higher numbers than the 30-day old lure (p = 0.0003). SPLAT-ME dollops thus continued to attract males, in spite of becoming relatively less attractive over time.

**Figure 5:**
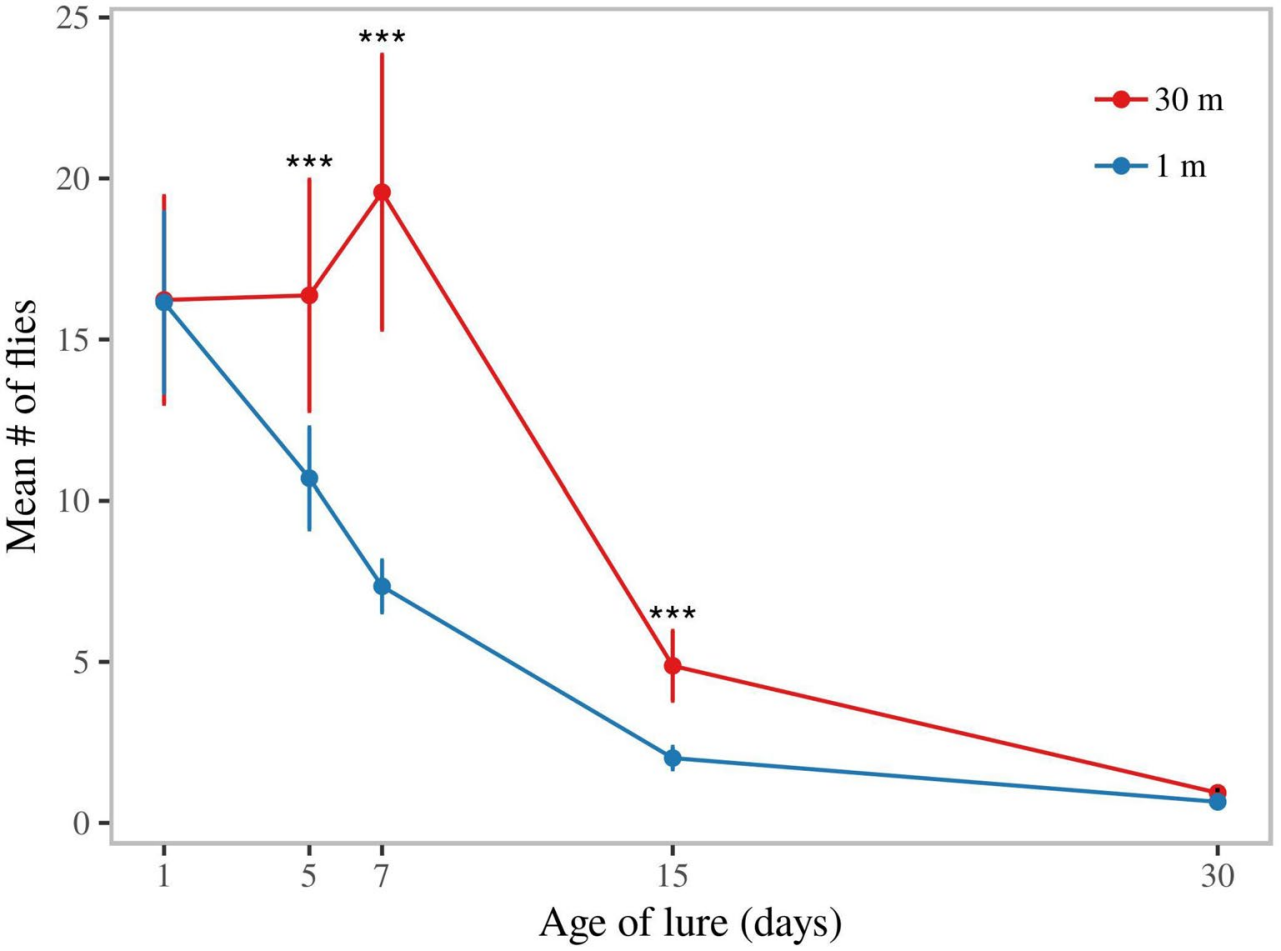
Field life of dollops containing methyl eugenol (ME). Capture of *B. dorsalis* males in traps baited with ME lures of different age (in days since application in orchard). Dollops of ME + spinosad were aged on mango leaves for 1, 5, 7, 15 and 30 days. Traps were either placed in proximity (at ~ 1 m distance and under the same canopy, i.e. in competition with each other), or at 30 m distance from each other.

Finally, the average capture rate in the experiment with traps spaced at 30 m was higher than when traps were placed in close proximity of each other (GLMM, poisson and lure age as random effect, p < 0.001; Fig. 5), indicating that traps at close range from each other competed for the same fruit flies that were locally present.

### Emergence from fruits indicates competitive displacement of *C. capitata* by *B. dorsalis*

**Figure 6:**
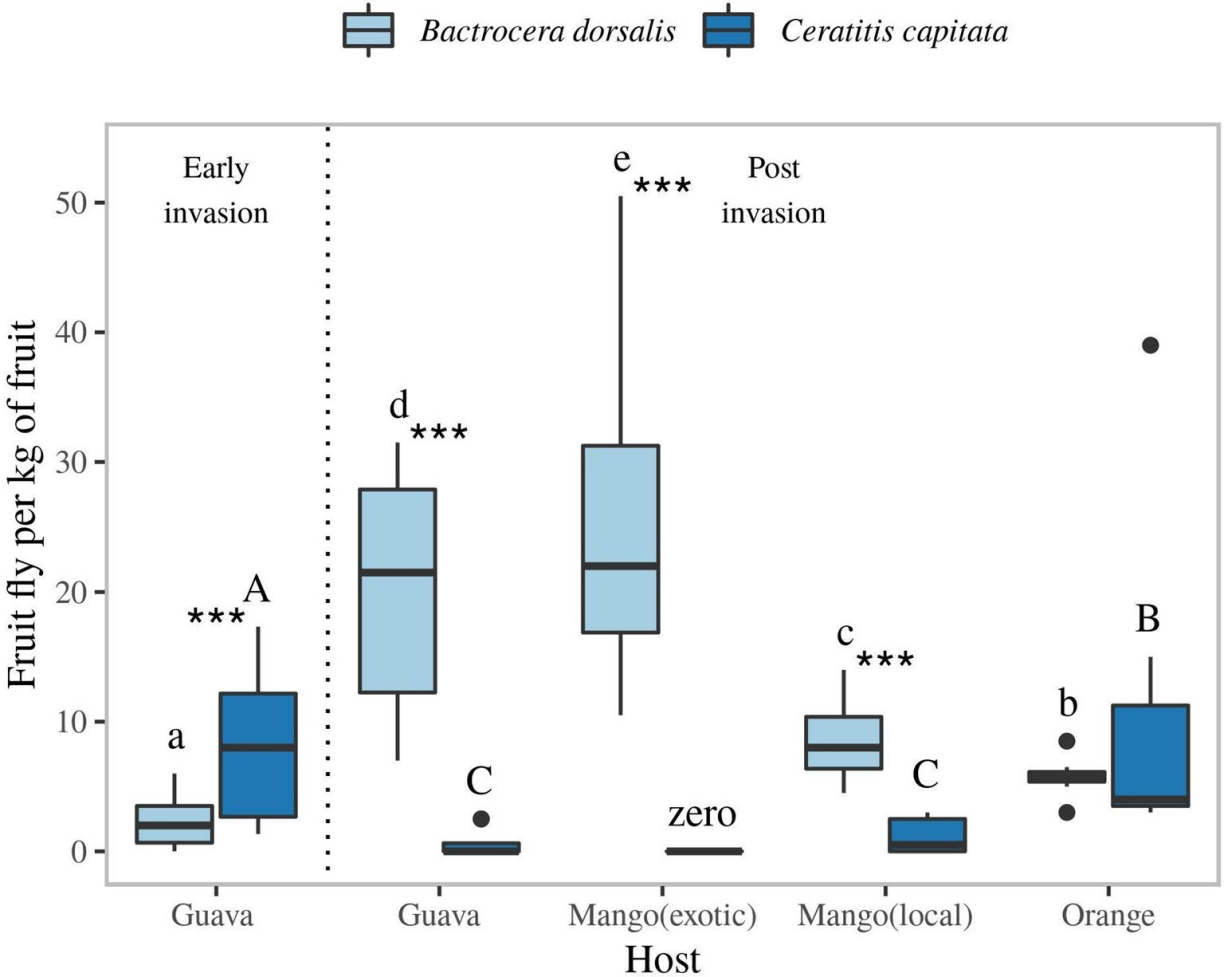
Number of adult *C. capitata* and *B. dorsalis* emerging from guava, mango (two cultivars), and orange collected in Arba-Minch. The left part of the graph shows emergence from fruits prior to *B. dorsalis* invasion (only in guava), whereas right part of the graph shows emergence after invasion of *B. dorsalis*. Tukey’s boxplots indicate the median, the 25 and 75 percentile boundaries (box), and 1.5 times the interquartile (IQ) range (whiskers). Dots represent outliers outside the 1.5 IQ range. Different letters/letter cases indicate a difference in fruit fly emergence per kg of fruit per host, with capital letters representing *C. capitata* and small letters representing *B. dorsalis*. Statistics were performed using a general linear model with a poisson distribution, n = 8, *** represents differences between species at p ≤ 0.001.

To assess potential competitive displacement of native *C. capitata*, we examined resource utilization by *C. capitata* and *B. dorsalis* through comparison of the number of emerging adults per kilo of fruit. Emergence rates of *B. dorsalis* and *C. capitata* followed opposite patterns: from fruits from which significantly more *B. dorsalis* emerged, significantly fewer *C. capitata* emerged (Fig. 6). Furthermore, significantly higher numbers of *C. capitata* emerged from guava at early stages of invasion of *B. dorsalis*, compared to post invasion. Conversely, *B. dorsalis* emergence was significantly higher in post invasion (after establishment) than in early invasion stages, which strongly indicates that *B. dorsalis* competitively displaced *C. capitata*.

### Capture efficiency of single or combined male lures

**Figure 7:**
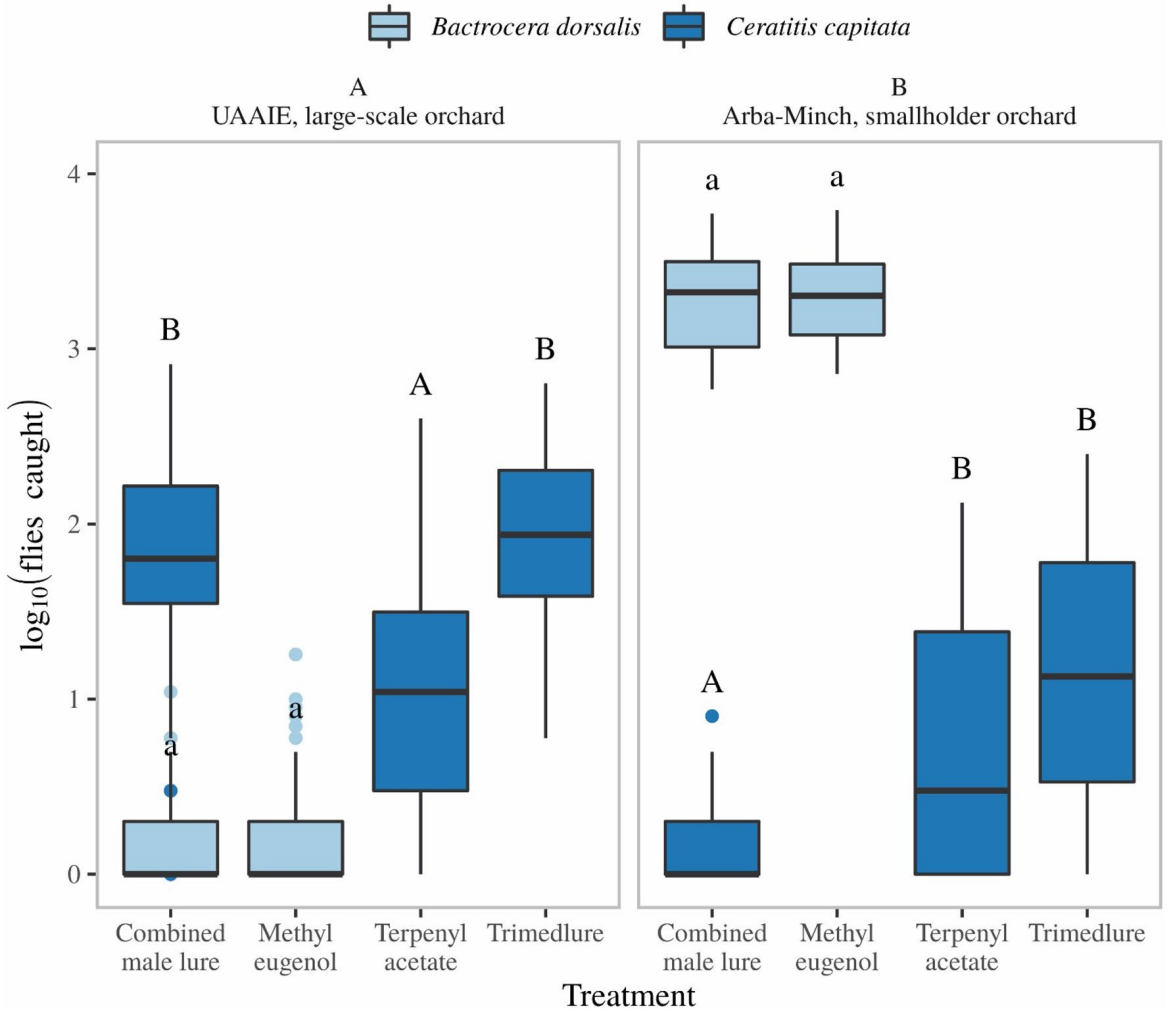
Comparison of catches of *B. dorsalis* and *C. capitata* with lures baited with different male attractants in both large-scale commercial orchards (UAAIE, A), and smallholder orchards (Arba-Minch, B). Statistics were performed using a generalized linear mixed model using a negative binomial distribution and by taking the variable “week” as random effect. Capital and lower-case letters are used to show statistically significant differences for different species, capital letters are used for *C. capitata* and small letters are used for *B. dorsalis*.

In the UAAIE large-scale farm, following suppression of the *B. dorsalis* population, the *C. capitata* population rebounded. Under these circumstances the combined male lure (CML) and TML captured similar numbers of *C. capitata,* while TA was less effective (Fig. 7A). Similarly, ME alone or in combination with the other male lures captured similar numbers of *B. dorsalis* (Fig. 7B). In smallholder farming settings in Arba-Minch, where no management procedures were in place, the catches of *B. dorsalis* were several orders of magnitude higher than that of *C. capitata* (n = 64, p < 0.001, Supporting information Fig. 2). Under these circumstances, combining TML with ME (CML) reduced the number of *C. capitata* caught compared to traps baited with TML or TA alone (Fig. 7B). In contrast, *B. dorsalis* capture with ME was unaffected in combination with TA and TML (Fig. 7B).

## Materials and methods

### Study areas

The study was conducted at two sites in Ethiopia. One of the sites is a large-scale commercial farming enterprise, the Upper Awash Agro Industry Enterprise (UAAIE), located 205 km east of Addis Ababa, at 8° 46′ 00.9ʺ N 39° 52′ 09.7ʺ E. The minimum temperature was 12 °C and maximum temperature was 35 °C, and humidity ranged between 32 and 62% RH. At UAAIE, the study was conducted in an irrigated guava (*Psidium guajava*) orchard of 55 ha, and contains fruits year-round. The second site, located 481 km south of Addis Ababa, at 6° 06′ 52.2ʺ N 37° 36′ 28.8ʺ E, included small scale mango plots of the Plant Health Clinic of Arba-Minch, and nearby small-scale farms. In Arba-Minch, the study was conducted in small orchards belonging to small-holders that cultivate mango (*Mangifera indica*) as the principal fruit crop. Mango fruit is harvested twice a year following the rainy seasons of March and September. Growers cultivate different varieties of mangoes (exotic and local), interspersed with other potential host of tephritids such as guava (*P. guajava*), papaya (*Carica papaya*), orange (*Citrus sinensis*) banana (*Musa* spp*.*), and avocado (*Persea americana*). Minimum and maximum temperature records were 14 °C and 34 °C, respectively, and humidity ranged between 61 and 98% RH.

### Intervention

Mixtures of different male lure formulations, ME (20% ME, 2% spinosad), ME (20%), TML (10% TML + 5% sugar + 2% spinosad), TA (10% TA + 5% sugar + 2% spinosad), and a combined male lure (CML, 20% ME + 10% TA + 10% TML + 5% sugar + 2% spinosad) were formulated in SPLAT MAT, (SPLAT-Specialized Pheromone and Lure Application Technology, ISCA Technologies, CA, USA) (MAT-male annihilation technique). SPLAT is a commercial slow-release matrix SPLAT and is a modified formulation from Atterholt et al.^26^. SPLAT has been used in pheromone-based technologies (e.g. mating disruption), kairomone-based technologies (e.g. attract and kill, or repellence), as well as sustainable release of semiochemical and control agents in numerous studies, including in the release of male lures for tephritids^27^. The waxes and oils used in SPLAT are biodegradable^26,27^.

At the large-scale farm three main techniques were used by the management to suppress *B. dorsalis* population throughout the entire farm, including M3 Fruit Fly Bait Station (Green trading cc, SA), GF-120 containing spinosad (DOW AgroSciences LLC, Indianapolis, IN) and orchard sanitation. GF-120 was diluted in water as recommended in the label 1:5 ratio of GF-120:water and sprayed with a hand-pumped back-pack sprayer using a conventional conical nozzle with 1–2 mm aperture. Approximately 25 ml/tree of the solution was sprayed on a regular schedule at 15 days interval at approximately 1.5 m on trees not bearing fruit. The M3 Fruit Fly Bait Stations were hung on trees at 4 m distances. The efficacy of the treatments (GF-120 & M3) were monitored using a variable number of traps (Easytrap (H14 cm X W9cm X D5cm J.p. Ros, INIA, Madrid) per ha baited with ME and malathion. We plotted the average fruit fly per trap per day (FTD) values of trap catches in the Guava orchard over 8 months (Fig. 1).

Following the suppression of *B. dorsalis*, and the resurgence of *C. capitata*, we rolled out treatments to assess how population levels of both species developed using SPLAT-MAT based methods. A total of 4 experimental blocks of guava were divided into 4 plots of ~ 1 ha each. Two of the plots in 3 of the blocks were treated with ME + spinosad, against *B. dorsalis*) dollops and the other two plots of these 3 blocks were treated with TA + TML + spinosad dollops (against *Ceratitis* species). Each plot received 360 g divided over 120 dollops. All plots in the fourth block were treated with dollops of ME alone without insecticide, which served as control plots. Dollops were applied in caps of water bottles and tied to branches of guava trees, which were removed once a week for assessing population levels. Four different monitoring traps (CML + spinosad, TML + spinosad, ME + spinosad & ME alone) were deployed in all plots and population levels were assessed each week. The experiment was conducted during 35 weeks between 2014-11-19 and 2015-07-22.

In small-scale farms between 2015-04-05 and 2016-07-04 we tested a ME-based attract-and-kill formulation for suppressing the dominant species *B. dorsalis*. Dollops of ME (20% ME, 2% spinosad) were applied in six plots, whereas dollops of ME (20%) without spinosad were applied in two control plots. Each plot was approximately 1 ha in size. Even though the plots consisted of many different potential host plants, such as papaya, orange and guava, the mango dominated the plots with between 80 and 147 trees per plot. A dollop (~ 3 g) was manually applied on a shaded leaf at the lower part of the canopy of each mango tree. Population levels of both species (*B. dorsalis* and *C. capitata*) were monitored weekly in each plot by deploying nine traps baited with CML (combined male lure, consisting of 20% ME + 10% TA + 10% TML + 5% sugar + 2% spinosad) for 24 h. Every month, treated leaves were removed, buried and replaced with fresh dollops.

### Mark release recapture

A mark release recapture (MRR) study was conducted in Arba-Minch between 2016-04-12 and 2016-08-25, in small-scale farming settings. To collect males for marking, modified traps were constructed (Fig. 3 middle inset), such that male flies could be collected with ME without them contacting the lure. Collected flies were immobilized by placing the buckets in iceboxes for 30–40 min and counted. Flies were marked by spraying one of two different colors (blue & green) of readmission ink (Blak-Ray UVP Inc, Upland California, USA). After marking, the flies were allowed to leave the bucket for one hour between 1500 and 1600 h. Flies that did not take off were counted and subtracted from the total number of flies released. The MRR experiment was designed in order to assess the magnitude of dispersal of the fruit flies. Eight traps were used in the release plot, while five traps were placed in each of seven other plots and five traps were placed as far away as 1 km from the release point to assess long range dispersal. Two colors were used to assess the dispersal of flies. A group of green-marked flies was released, followed by blue-marked flies three days later. Seventy-two hours after the release of the green-marked flies, methyl eugenol-baits were deployed to recapture dispersed flies. We argued that if we would place ME traps directly after release, dispersal would have been inhibited. So, the green marked flies (released before traps were deployed) served for getting a potentially more accurate measure of the dispersion rate, giving flies the time to disperse first, while the blue marked flies would, we assumed, result in a higher recapture rate. The releases of marked flies were repeated three times with a month interval between 2016-08-25 and 2016-04-22. For each set of release trap captures were analyzed every 5 days for 30 days. Colors of marked flies were identified using an ultraviolet flashlight torch (TOPCOM, TP-7312DUV, Zhejiang, China). Experiments were performed when no other experiments were ongoing in the area.

### Longevity

For estimates of the field life of ME + spinosad, experiments were conducted in Arba Minch between 2015-09-18 and 2016-04-20. Dollops of approximately 3 g of SPLAT containing 20% ME & 2% spinosad were aged on mango leaves in the lower shaded part of a canopy of a mango tree for 1, 7, 15 and 30 days prior to the experiment. At the start of the experiment, leaves containing treatments from each application date were detached from the mango tree and placed inside modified traps, which consisted of a transparent water bottle (1.5 L) cut in half and inverted, with two fly entry holes (~ 7 mm, diam.). The fruit flies could easily enter through the holes, but we limited the entry hole to 7 mm so that flies did not readily exit the traps before the spinosad took effect. For the sake of determining the effect of aging on the relative attractiveness, baited traps containing the differently aged treatments were hung in proximity of each other (at 1 m distance, under the same mango tree canopy). In a separate experiment, we assessed how long a lure could be effective in attracting and suppressing the male population. In this experiment we placed baited traps containing the differently aged treatments at 30 m distance from each other to get a relatively independent measure of the rate of attraction of each differently aged lure. In both experiments trap position was rotated between replicates. Captures were evaluated after 24 h.

### Fruit sampling and competitive displacement

To determine potential competitive displacement and resource partitioning of *C. capitata*, ripe fruits from, guava, *P. guajava*, orange, *C. sinensis* and exotic & local varieties of mango, *M. indica* were randomly sampled from intervention treated plot in Arba-Minch. Sampling was performed from March to June 2015. Trees from each host fruit were randomly selected and variable numbers of fruits with oviposition marks were collected depending on availability and fruiting season. In total, 1440 local mango, 1440 guava, 624 orange and 360 exotic mango fruits were sampled and weighed. Samples were transferred to the laboratory at Arba-Minch plant health clinic and placed in plastic containers (12 × 22 × 30 cm) on a dry sand layer (1–2 cm deep). Depending on the size of the fruits, 5–10 fruits were placed in each plastic container. Fruits were transferred to other plastic containers every 3 days. Pupae recovered from sands were transferred to bug dorm cages (30 × 30 × 30 cm) for identification of emerging fruit fly adults.

### Lure combination & capture efficiency

Attractiveness of the combined male lure (CML, which contains 20% ME + 10% TA + 10% TML + 5% sugar + 2% spinosad) to *B. dorsalis* and *C. capitata* flies was compared with its individual components: ME (20% ME + 2% spinosad), TML (10% TML + 5% sugar + 2% spinosad) and TA (10% TA + 5% sugar + 2% spinosad) to test if the combinations of male attractants would result in different catch numbers compared to single lures. For this experiment, two types of traps were used. In the smallholder farming setting a modified transparent water bottle trap (1.5 L) with two fly entry openings in the upper half of the bottle (~ 7 mm diam.) was used. In the large-scale farm, we used a commercially available trap, Easytrap. Traps were baited with a 3 g dollop of either ME, TML, TA or CML. Four traps, each containing one of the dollops, were hung at 30 m distance from each other. This was replicated in four plots at Arba-Minch and four plots at UAAIE (a plot is ~ 1 ha). As the *B. dorsalis* population was high in Arba-Minch, we used the larger modified water bottle traps, and trap catches were counted daily. At UAAIE traps were serviced once a week. The experiment was conducted between 2015-02-14 and 2015-07-02.

### Data analysis

For determining the effect of intervention using male lures on population levels, trap capture values were normalized by dividing catches for each individual trap at each date with the average catches during the first two weeks of the intervention, since no separation between control and treatment could be observed no further analysis was performed.

For the mark and release study, a correlation analysis was made using linear models to correlate the distance in log10(meter) from release point and the average trap catch of marked and resident flies. For visualization purposes a map was produced using the average trap catch average per location and the package ggmap, a wrapper for ggplot2 was used for accessing the google API and downloading the map^28^, temperature and wind data was gathered using the package WeatherData^29^, where a temperature set was filtered for 12:00 h local time. The map was annotated with each of the orchards and trap position and concentric circles representing the distances from release point. Temperature data was plotted using a violin plot with a dotted line representing the average, and wind direction using a radial plot where the number and strength of each wind vector was plotted.

Lure longevity was assessed for traps placed at 30 m distance as well as in close proximity (1 m) and for each lure ‘age’ using a generalized linear mixed model (glm) fitted with a negative binomial distribution (study week as random effect), this was followed by a Tukey’s all-pairwise comparison to determine differences between differently aged lures. Akaike’s information criteria (AIC) values as well as patterns of residual values were used to determine normality and the best fitting model/distribution.

A generalized linear model (glm) fitted with a poisson distribution was used to compare (a) for each fruit the number of emerging *B. dorsalis* relative to *C. capitata* adult per fruit sample, and (b) second, compare the emerging adult fly species across the fruit species. Differences were sorted using post-hoc comparisons (multcomp::glht).

To analyze capture efficiency and combination of lures, a generalized linear mixed-effects model (glmm) fitted with a negative binomial was used. The theta value was calculated from a zero model. For models using glmm the package lme4^30^ was used, while stats package was used for glm. Post hoc tests were done using package multcomp^31^ and Tukey’s all-pairwise comparisons. All graphs were made using ggplot2^32^ and all analysis were done using R (version 3.53.12^33^).

### Ethics declarations

This article does not involve any human and/or animal participants.

## Discussion

Damage caused by native tephritid fruit flies has always been high in sub-Saharan Africa^19^. However, the arrival of the invasive pest *B. dorsalis*, seriously aggravated the problem, with up to a 100% damage on certain fruit crops in selected areas^34^. Management tools tailored to local parameters need to be developed and evaluated^35^. This study evaluated several parameters that are relevant for African farming settings, that impact the effectiveness of male lures in suppressing fruit fly populations and securing fruit production. These include the scale of production, potential for competitive release, field longevity of the male lure, suitability of host fruits, and the dispersive ability of the pest.

### Intervention large-scale farm and smallholder settings

Since the invasion of *B. dorsalis* at UAAIE at around 2010, the species rapidly replaced *Ceratitis* species as main pest. At the large-scale farm, UAAIE, a combination of management methods successfully reduced the invasive pest *B. dorsalis* within 8 months of intensive control measures. Their efforts anticipated our trial using SPLAT-ME, which could therefore not be carried out. Nevertheless, the data collected during the intervention by UAAIE management demonstrates that it is possible to suppress this highly invasive pest. Yet, although the suppression reduced *B. dorsalis*, it caused a resurgence of native *C. capitata* within a few months, a species that was rarely caught post invasion of *B. dorsalis*. This indicates that removal of one species (*B. dorsalis*) can cause competitive release of the replaced species (*C. capitata*), a phenomenon well described in other production systems^36,37^. Clearly, in the presence of a fruit-fly guild, combinations of methods need to be deployed, particularly when the methods target the dominant species almost exclusively (*B. dorsalis*).

Whereas we deployed lures that target multiple species, *C. capitata* appeared not to be suppressed much during the trial. There may be several reasons for this. First, trimedlure is a much less potent attractant for *C. capitata* than methyl eugenol for *B. dorsalis*. In addition, unlike *B. dorsalis* which readily feed on ME^38^, *Ceratitis* spp are not known to feed on male lures^39^. The addition of a phagostimulant (sugar) to the bait was aimed at increasing feeding and thus lethal spinosad dose uptake, but perhaps in part due to the weaker attractiveness of the lure this did not suppress the population sufficiently. Because of the inability to suppress fruit flies altogether, the guava orchard was uprooted in 2016.

Intervention trials in smallholder farms in Arba Minch did not result in suppression of the target pest *B. dorsalis*. We infer from our mark-release-recapture data that a main contributing factor to this failure was the high likelihood of *B. dorsalis* to disperse the distance of many smallholder plots, and the availability of several hosts in smallholder settings (see below). It is also important to note that in the smallholder farming area no other concerted management efforts were rolled out against fruit flies.

### Mark-release-recapture

For any intervention method it is critical to know not only its efficacy in suppressing the resident population, but also the extent of immigration of the pest from neighboring areas^40^. This is particularly true for mating disruption-based methods, in which the damaging sex (the female) is only indirectly affected by removing the non-damaging sex (male)^41^. In such cases, the efficacy is dependent on the extent of males or mated females moving from neighboring untreated areas into the treated area. Mark-release-recapture studies are good tools to assess this factor. For tephritid fruit flies mark-release-recapture studies have been performed in the past, and these show that flies can disperse over long distances, from 11 km up to 19 km for *B. dorsalis*^42,43^. However, these were done in monoculture settings with moderate winds, compared to ‘calm’ weather conditions (very low wind) and complex culture systems that characterize smallholder-farming in East Africa. Furthermore, the immigration factor is critical in establishing the scale of intervention needed to ensure effective population control, which quickly exceeds the small farm size of smallholder farmers (typically less than 1 ha). Under the conditions tested here, including the high populations levels of *B. dorsalis*, the low winds and indicated temperatures, *B. dorsalis* readily disperses hundreds of meters in smallholder farming settings, even though fruit and food were abundantly present at and close to the release point. Considering the fact that recapture rates naturally drop with distance (Fig. 4, fewer sampling points per surface area with increased distance from the release points), the relative recapture rate being as high as 60% (of the total recaptured flies) over 300 m and around 8% at over 1000 m are likely a gross underestimate of the actual fraction of flies engaging in long-range dispersion. In addition, the figures may further underestimate natural dispersion, because the capturing and marking may have affected the flies’ fitness to disperse (although low, 11% remained in the release bucket, possibly due to capturing and crowding in the bucket before release) and possibly the reduced likelihood to be recaptured using the same trap and lure (possible negative associative learning). A significant portion of male *B. dorsalis* thus disperses across the boundaries of a few to many smallholder plots (See Fig. 3, light-shaded areas indicating farm boundaries). This is further underlined by the fact that recapture rates of green marked flies, which were released 3 d prior to placement of the traps, were lower than those released at the same time of trap placement, particularly for traps within a 500 m distance from the release point, which may partly indicate that flies rapidly disperse outside the release area (Fig. 3). Thus, in smallholders farming areas in East Africa, fly management using male annihilation, and likely any other technique, require concerted efforts by the farmers and area-wide management strategies^44–46^. It also implies that techniques other than male annihilation, and more focused on reducing the female population, may be more appropriate for such small farm sizes. Further research should evaluate the relative dispersive capabilities of female flies, relative to males. This has not been studied in any previous study, while the impact of dispersing females is much more significant from a population dynamics and pest control perspective.

### Lure longevity and trap spacing

An important component for developing a new, sustainable lure for use in pest control is its field life. Prolonged attractiveness and the lethality reduce the need for frequent labor-intensive and expensive reapplications. In our experiments, lures indeed lost their attractiveness over time, even though ME is the most powerful male lure for tephritid species and known to attract certain species from distances, even at low doses^47^. When placed in a competitive setting (in close proximity to each other), trap catch gradually declined with ageing of the lure. A decline in attractiveness of ME lures was also observed in studies with other insect species^48,49^. Fresh lures release higher amounts of ME than aged lures and are thus more attractive^50,51^, as there seems no upper boundary of ME release above which the compound becomes repellent^52^. However, this difference may only be noticeable when traps are sampling from the same local population and thus are ‘competing’ for the same flies (as in our 1-m spaced traps). Indeed, a recent study also indicated that efficacy does not necessarily increase with application density of SPLAT-MAT-ME dollops, and that above a saturation level efficacy of attract-and-kill no longer increases, or even decreases^53^. The same study also did not find a clear decrease in efficacy of lures of 1-d old and 14-d old lures. This is similar to what we found here, as capture rates of differently aged lures in our 30-m spaced traps experiments showed a consistent decline in capture rates of lures of more than 7 days old. Note also that the longevity of lures measured here may be influenced by climatic factors and thus cannot directly be translated to other climate zones^54^. The results also suggest that, although ME is a powerful attractant, the range of attraction of ME may be less than 30 m. As this is an indirect inference from the data, however, detailed studies are needed to examine the exact range of attraction.

It should be noted that, although the captures declined with ageing, this may also have been in part due to degradation of Spinosad in the bait and not only because of loss of methyl eugenol from the dollop, as flies exit traps that do not contain Spinosad-laced baits. Regardless of the decline, attract-and-kill may still work well over prolonged time if dollops are applied at higher density. With ageing the lower range of attraction of dollops with attenuated ME strengths could be compensated for by a higher application density. From an application perspective, further studies should aim at estimating the range of attraction, and ways to minimize decline in attractiveness of dollops over time and estimate the economic viability of these strategies.

### Competitive displacement of *C. capitata* by the invasive *B. dorsalis*

Species with a similar ecological niche can coexist through resource partitioning. The strength of interspecific competition is dependent on the variety of available host fruits that competing pests can utilize^55^. The intercropping of various hosts in the smallholder orchards likely provides opportunities for partition resources of competing Tephritidae species. Interestingly, in smallholder farms the invasive *B. dorsalis* was mainly collected from recently introduced mango varieties such as ‘Kent’ and ‘Tommy Atkins’, whereas *C. capitata* flies were mainly collected from local mango and orange, from which few *B. dorsalis* emerged. This suggests that *B. dorsalis* competitive displaced *C. capitata* fly onto fruit less preferred by the first (Fig. 6). The complex array of different host species, and of varieties within a species, that typifies smallholder farming plots in East Africa may create more competition-free niches that allow for coexistence of several tephritid species in the same location. In fact, in spite of the high infestation levels of *B. dorsalis*, several other species besides *C. capitata*, were continuously captured in smallholder plots, albeit in low numbers. In contrast, the various *Ceratitis* spp. that challenged production in the large monoculture guava plots at UAAIE prior to *B. dorsalis* invasion^18^, completely disappeared from monitoring traps placed in guava at the start of intervention in 2013, except for *C. capitata*. This may be circumstantial evidence for niche differentiation in the smallholder plots, and absence thereof in the large-scale plots. In La Réunion, despite niche overlap with other tephritid pests, *C. capitata* was found to uniquely exploit certain hosts^56,57^. Similarly, Vargas et al.^4^, found that in spite of competitive displacement by *B. dorsalis*, *C. capitata* remained dominant in coffee plantations, which is considered a non-host for *B. dorsalis*.

### Interaction of male lures and trap interference

As our field observations indicated that suppression of one pest species can lead to a resurgence of another pest species that occupies the same niche, fruit fly management should target all species in the guild to achieve an effective crop protection. Since different tephritid species are attracted to different male attractants, it would seem logical and economical to combine lures in the same dispenser. We verified possible interactions due to combining such lures (TA and TML, attractant of *C. capitata*, among other species, and ME attractant of *B. dorsalis*). The efficacy of combining male specific lures has been reported to be variable, depending on population density of the interacting species and locality^58^. As no lure interaction was noted in large-scale farms, we suspect that the very high populations of *B. dorsalis* in smallholder farms suppressed trap entry and catches of *Ceratitis*. Indeed, in Arba-Minch many *B. dorsalis* males were seen on the outside of the trap, which probably interfered with trap entry of *C. capitata*, particularly since ME is a much stronger male attractant for *B. dorsalis* than TML is for *C. capitata*. We therefore cannot confirm that different male lures influence trap catches of either species when placed in the same trap, which has been reported in the literature. For instance, Vargas et al.^58^ reported that traps baited with the combination of 50% ME and 50% cuelure (another male lure attractive to certain tephritid species) were equally attractive to *Zeugodacus cucurbitae* as traps baited with cuelure alone, while other reports showed negative or synergistic interactions between these^59,60^.

## Conclusion

We demonstrated that in large-scale farming settings in East Africa, male lures in combination with other semiochemical management tools can effectively suppress the invasive *B. dorsalis*. However, the risk of resurgence of native fruit fly pests, including *C. capitata*, illustrates the need for combinatorial tools that target multiple Tephritidae species.

In our smallholder orchards, where the native fruit fly *C. capitata* had been largely displaced by *B. dorsalis*, application of ME-based attract-and-kill technique did not suppress *B. dorsalis* to manageable levels, in part due to the species’ long dispersive range. Controlling tephritid pests in a smallholder-farming setting thus requires concerted efforts from farming communities to achieve areawide management. Further, the complex and diverse cropping systems in smallholder farms may offer competition-free space for a more diverse fruit fly guild, thereby increasing the risk for competitive release and requiring intervention that targets multiple species.

Whereas previously pest issues were ‘resolved’ using broad-spectrum insecticides, the current roll-back of these products offers opportunities for novel and more sustainable alternatives. However, these methods are almost invariably more knowledge-intensive and require studies to assess if, how, and under which conditions, they can provide sustainable control and secure food production and economic growth. We hope that the current study contributed to this, particularly for African smallholder and large-scale farming settings.

## Supplementary Information

Supplementary Information

## References

[1] Duyck P-F, David P, Junod G, Brunel C, Dupont R, Quilici S (2006). Importance of competition mechanisms in successive invasions by polyphagous tephritids in La Réunion. Ecology.

[2] Duyck P-F, David P, Quilici S (2004). A review of relationships between interspecific competition and invasions in fruit flies (Diptera: Tephritidae). Ecol. Entomol..

[3] Ekesi S, Billah MK, Nderitu PW, Lux SA, Rwomushana I (2009). Evidence for competitive displacement of *Ceratitis cosyra* by the invasive fruit fly *Bactrocera invadens* (Diptera: Tephritidae) on mango and mechanisms contributing to the displacement. J. Econ. Entomol..

[4] Vargas RI, Walsh WA, Nishida T (1995). Colonization of newly planted coffee fields: Dominance of Mediterranean fruit fly over Oriental fruit fly (Diptera: Tephritidae). J. Econ. Entomol..

[5] Malacrida AR, Gomulski LM, Bonizzoni M, Bertin S, Gasperi G, Guglielmino CR (2006). Globalization and fruit fly invasion and expansion: the medfly paradigm. Genetica.

[6] Poyet M, Roux VL, Gibert P, Meirland A, Prévost G, Eslin P (2015). The wide potential trophic niche of the Asiatic fruit fly *Drosophila suzukii*: the key of its invasion success in Temperate Europe?. PLoS ONE.

[7] Nyamukondiwa C, Kleynhans E, Terblanche JS (2010). Phenotypic plasticity of thermal tolerance contributes to the invasion potential of Mediterranean fruit flies (*Ceratitis capitata*). Ecol. Entomol..

[8] Reitz SR, Trumble JT (2002). Competitive displacement among insects and arachnids. Annu. Rev. Entomol..

[9] Sakai AK, Allendorf FW, Holt JS, Lodge DM, Molofsky J, With KA (2001). The population biology of invasive species. Annu. Rev. Ecol. Syst..

[10] Manrakhan A, Venter JH, Hattingh V (2015). The progressive invasion of *Bactrocera dorsalis* (Diptera: Tephritidae) in South Africa. Biol. Invas..

[11] Shimizu Y, Kohama T, Uesato T, Matsuyama T, Yamagishi M (2007). Invasion of solanum fruit fly *Bactrocera latifrons*(Diptera: Tephritidae) to Yonaguni Island, Okinawa Prefecture, Japan. Appl. Entomol. Zool..

[12] Perrings C, Dehnen-Schmutz K, Touza J, Williamson M (2005). How to manage biological invasions under globalization. Trends Ecol. Evol..

[13] De Meyer, M., Mohamed, S. & White, I.M. Invasive fruit fly pests in Africa: A diagnostic tool and information reference for the four Asian species of fruit fly (Diptera, Tephritidae) that have become accidentally established as pests in Africa, including the Indian Ocean Islands. Online at: http://www.africamuseum.be/fruitfly/AfroAsia.htm [accessed 12 September 2017] (2017).

[14] Lux SA, Copeland RS, White IM, Manrakhan A, Billah MK (2003). A new invasive fruit fly species from the *Bactrocera dorsalis*(Hendel) group detected in East Africa. Int. J. Trop. Ins. Sci..

[15] Ekesi S, Nderitu PW, Rwomushana I (2006). Field infestation, life history and demographic parameters of the fruit fly *Bactrocera invadens* (Diptera: Tephritidae) in Africa. Bull. Entomol. Res..

[16] Goergen G, Vayssières JF, Gnanvossou D, Tindo M (2011). *Bactrocera invadens* (Diptera: Tephritidae), a new invasive fruit fly pest for the Afrotropical region: Host plant range and distribution in West and Central Africa. Env. Entomol..

[17] Vayssières JF, Korie S, Ayegnon D (2009). Correlation of fruit fly (Diptera Tephritidae) infestation of major mango cultivars in Borgou (Benin) with abiotic and biotic factors and assessment of damage. Crop Protect..

[18] Dessie, B. Species composition, population dynamics and relative economic importance of fruit flies (Diptera: Tephritoidea) on guava, mango and citrus at Upper Awash River Valley*. PhD Dissertation*, Haramaya University, Ethiopia (2014).

[19] Ekesi S, Meyer MD, Mohamed SA, Virgilio M, Borgemeister C (2016). Taxonomy, ecology, and management of native and exotic fruit fly species in Africa. Annu. Rev. Entomol..

[20] Tan KH, Nishida R, Jang EB, Shelly TE, Shelly T, Epsky N, Jang E, Reyes-Flores J, Vargas R (2014). Pheromones, male lures, and trapping of tephritid fruit flies. Trapping and the Detection, Control, and Regulation of Tephritid Fruit Flies.

[21] Vargas RI, Mau RFL, Stark JD, Piñero JC, Leblanc L, Souder SK (2010). Evaluation of methyl eugenol and cue-lure traps with solid lure and insecticide dispensers for fruit fly monitoring and male annihilation in the Hawaii area wide pest management program. J. Econ. Entomol..

[22] Koyama J, Teruya T, Tanaka K (1984). Eradication of the oriental fruit fly (Diptera: Tephritidae) from the Okinawa Islands by a male annihilation method. J. Econ. Entomol..

[23] Ndlela S, Mohamed S, Ndegwa PN, Ong'Amo GO, Ekesi S (2016). Male annihilation technique using methyl eugenol for field suppression of Bactrocera dorsalis (Hendel) (Diptera: Tephritidae) on mango in Kenya. Afr. Entomol..

[24] Hanna, R., Gnanvossou, D., Grout, T. (2008) Male annihilation technique (MAT) in eliminating *B. invadens* in northern Bénin. Fighting Fruit and Vegetable Flies Regionally in Western Africa.

[25] Vargas RI, Souder SK, Mackey B, Cook P, Morse JG, Stark JD (2012). Field trials of solid triple lure and insecticide dispensers for detection and male annihilation of Ceratitis capitata (Wiedemann), Bactrocera dorsalis (Hendel) and Bactrocera cucurbitae (Coquillett) (Diptera: Tephritidae) in Hawaii. J. Econ. Entomol.

[26] Atterholt CA, Delwiche MJ, Rice RE, Krochta JM (1999). Controlled release of insect sex pheromones from paraffin wax and emulsions. J. Cont. Rel..

[27] Vargas RI, Stark JD, Hertlein M, Mafra Neto A, Coler R, Piñero JC (2008). Evaluation of SPLAT with spinosad and methyl eugenol or cue-lure for “attract-and-kill” of oriental and melon fruit flies (Diptera: Tephritidae) in Hawaii. J. Econ. Entomol.

[28] Kahle D, Wickham H (2013). ggmap: Spatial visualization with ggplot2. R Journal.

[29] Narasimhan, R. weatherData: Get Weather Data from the Web. R package version 0.4.5. (2016) http://ram-n.github.io/weatherData/ [Accessed November 2018]

[30] Bates D, Maechler M, Bolker B, Walker S (2015). Fitting linear mixed-effects models using lme4. J. Stat. Soft..

[31] Hothorn T, Bretz P, Westfall P (2008). Simultaneous inference in general parametric models. Biometr. J..

[32] Wickham, H. ggplot2 (Version: 3:0:0): Elegant Graphics for Data Analysis. *J Stat Softw* 35:65–88. URL: http://ggplot2.tidyverse.org (2010).

[33] R Core Team, R (2018) A language and environment for statistical computing. Vienna, Austria, *R Foundation for Statistical Computing*. URL: http://www.R-project.org/.

[34] Vayssières JF, Korie S, Coulibaly O, Temple L, Boueyi SP (2008). The mango tree in central and northern Benin: Cultivar inventory, yield assessment, infested stages and loss due to fruit flies (Diptera Tephritidae). Fruits.

[35] Aluja M, Ordano M, Guillén L, Rull J (2012). Understanding long-term fruit fly (Diptera: Tephritidae) population dynamics: Implications for areawide management. J. Econ. Entomol..

[36] LeBrun EG, Tillberg CV, Suarez AV, Folgarait PJ, Smith CR, Holway DA (2007). An experimental study of competition between fire ants and Argentine ants in their native range. Ecology.

[37] Sultana S, Baumgartner JB, Dominiak BC, Royer JE, Beaumont LJ (2020). Impacts of climate change on high priority fruit fly species in Australia. PLoS ONE.

[38] Shelly TE (1994). Consumption of methyl eugenol by male Bactrocera dorsalis (Diptera: Tephritidae): Low incidence of repeat feeding. Ann. Entomol. Soc. Am..

[39] Shelly TE (2001). Exposure to α-copaene and α-copaene-containing oils enhances mating success of male Mediterranean fruit flies (Diptera: Tephritidae). Ann. Entomol. Soc. Am..

[40] Mack RN, Simberloff D, Lonsdale WM, Evans H, Clout M, Bazzaz FA (2000). Biotic invasions: Causes, epidemiology, global consequences, and control. Ecol. Appl..

[41] Miller JR, Gut LJ (2015). Mating disruption for the 21st century: matching technology with mechanism. Environ. Entomol..

[42] Froerer KM, Peck SL, Mcquate GT, Vargas RI, Jang EB, Mcinnis DO (2010). Long-distance movement of *Bactrocera dorsalis* (Diptera: Tephritidae) in Puna, Hawaii: How far can they go?. Am. Entomol..

[43] Shelly T, Nishimoto J, Diaz A, Leathers J, War M, Shoemaker R, Al-Zubaidy M, Joseph D (2010). Capture probability of released males of two *Bactrocera* species (Diptera: Tephritidae) in detection traps in California. J. Econ. Entomol..

[44] Lloyd AC, Hamacek EL, Kopittke RA, Peek T, Wyatt PM, Neale CJ, Eelkema M, Gu H (2010). Area-wide management of fruit flies (Diptera: Tephritidae) in the Central Burnett district of Queensland, Australia. Crop Prot..

[45] Vargas RI, Piñero JC, Leblanc L, Manoukis NC, Mau RF (2016). Area-wide management of fruit flies (Diptera: Tephritidae) in Hawaii. Fruit Fly Research and Development in Africa-Towards a Sustainable Management Strategy to Improve Horticulture.

[46] Vreysen MJ, Robinson AS, Hendrichs J, Kenmore P (2007). Area-wide integrated pest management (AW-IPM): principles, practice and prospects. Area-wide control of insect pests.

[47] Roomi MW, Abbas T, Shah AH, Robina S, Qureshi SA, Hussain SS (1993). Control of fruit-flies (*Dacus* spp.) by attractants of plant origin. Anzeiger für Schädlingskunde Pflanzenschutz Umweltschutz.

[48] Broughton S, Rahman T (2017). Evaluation of lures and traps for male and female monitoring of Mediterranean fruit fly in pome and stone fruit. J. Appl. Entomol..

[49] Suckling DM, Stringer LD, Kean JM, Lo PL, Bell V, Walker JT (2014). Spatial analysis of mass trapping: How close is close enough?. Pest Manag. Sci..

[50] Vargas RI, Leblanc L, Pinero JC, Hoffman KM, Shelly T, Epsky N, Jang EB, Reyes-Flores J, Vargas R (2014). Male annihilation, past, present, and future. Trapping and the Detection, Control, and REGULATION of Tephritid Fruit Flies.

[51] Vargas RI, Souder SK, Nkomo E, Cook PJ, Mackey B, Stark JD (2015). Weathering and chemical degradation of methyl eugenol and raspberry ketone solid dispensers for detection, monitoring, and male annihilation of *Bactrocera dorsalis* and *Bactrocera cucurbitae* (Diptera: Tephritidae) in Hawaii. J. Econ. Entomol..

[52] Steiner LF (1952). Methyl eugenol as an attractant for oriental fruit fly. J. Econ. Entomol..

[53] Manoukis NC, Vargas RI, Carvalho L, Fezza T, Wilson S, Collier T (2019). A field test on the effectiveness of male annihilation technique against *Bactrocera dorsalis* (Diptera: Tephritidae) at varying application densities. PLoS ONE.

[54] Jang EB, Dowell RV, Manoukis NC (2017). Mark-release-recapture experiments on the effectiveness of methyl eugenol–spinosad male annihilation technique against an invading population of Bactrocera dorsalis. Proc. Hawaii Entomol. Soc..

[55] Kaplan I, Denno RF (2007). Interspecific interactions in phytophagous insects revisited: A quantitative assessment of competition theory. Ecol. Lett..

[56] Duyck P-F, David P, Pavoine S, Quilici S (2008). Can host-range allow niche differentiation of invasive polyphagous fruit flies (Diptera: Tephritidae) in La Réunion?. Ecol. Entomol..

[57] Duyck PF, David P, Quilici S (2006). Climatic niche partitioning following successive invasions by fruit flies in La Réunion. J. Anim. Ecol..

[58] Vargas RI, Stark JD, Kido MH, Ketter HM, Whitehand LC (2000). Methyl eugenol and cue-lure traps for suppression of Male Oriental fruit flies and melon flies (Diptera: Tephritidae) in Hawaii: Effects of lure mixtures and weathering. J. Econ. Entomol.

[59] Royer JE, Mayer DG (2017). Combining cue-lure and methyl eugenol in traps significantly decreases catches of most Bactrocera, Zeugodacus and Dacus species (Diptera: Tephritidae: Dacinae) in Australia and Papua New Guinea. J. Econ. Entomol..

[60] Shelly TE, Pahio E, Edu J (2004). Synergistic and inhibitory interactions between methyl eugenol and cue lure influence trap catch of male fruit flies, *Bactrocera dorsalis* (Hendel) and *B. cucurbitae* (Diptera: Tephritidae). Florida Entomol..

