# Supplementary material for: Dispersal and competitive release affect the management of native and invasive tephritid fruit flies in large and smallholder farms in Ethiopia
Source: Sci Rep. 2021 Jan 29;11:2690. doi: 10.1038/s41598-020-80151-1 (PMC7846734; doi:10.1038/s41598-020-80151-1)
Supplement: Supplementary file 1 — Supplementary Information [file 41598_2020_80151_MOESM1_ESM.docx]

Dispersal and competitive release affect the management of native and invasive tephritid fruit flies in large and smallholder farms in Ethiopia.

Tibebe Dejene Biasazin^1,2^, Tadiwos W Wondimu ^2^, Sebastian Larsson Herrera ^1^, Mattias Larsson ^1^, Agenor Mafra-Neto ^3^, Yitbarek W Gessese^2^, Teun Dekker ^1^


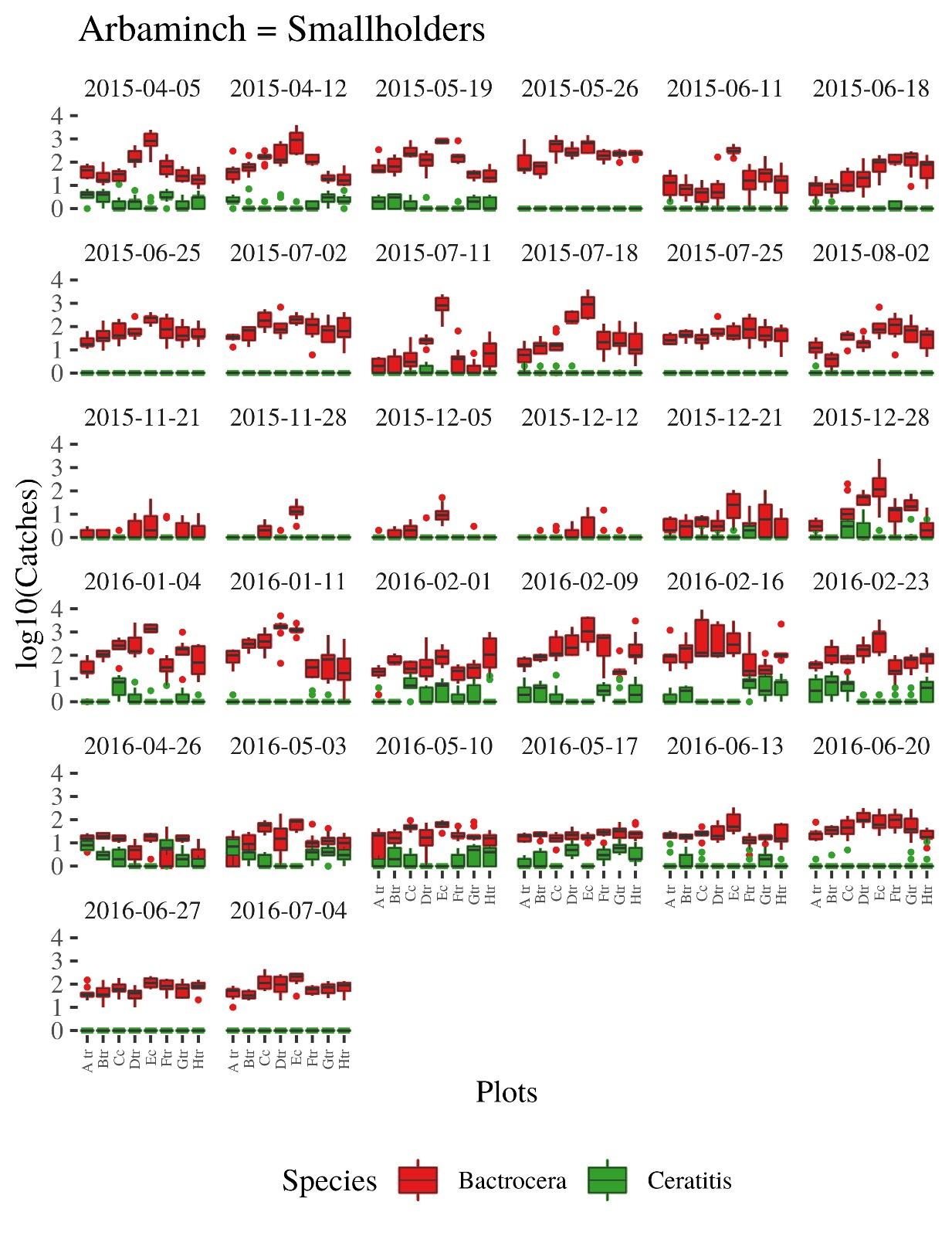


Supporting information Figure 1. Mean number of *B. dorsalis* (red) and *C. capitata* (green) flies captured in the treatment and control plots of smallholder orchards throughout the intervention period. (Atr, Btr, Dtr, Ftr, Gtr, and Htr are treated plots while Cc and Ec are control plots). Graphs were made using ggplot2 (Wickham, 2010), URL: <http://ggplot2.tidyverse.org>. using R (version 3.53.12, R Core Team, 2018), URL: <http://www.R-project.org/>.


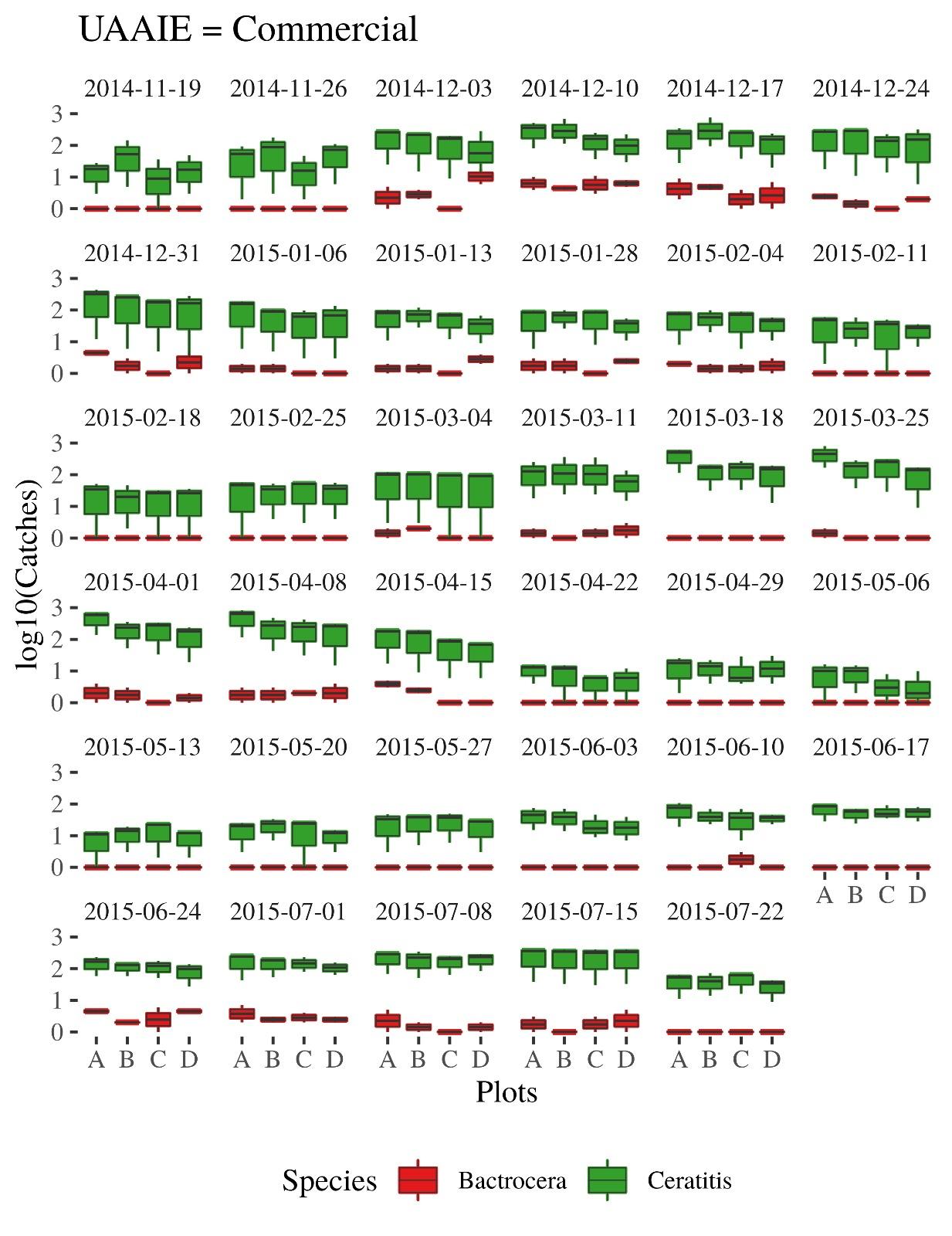


Supporting information Figure 2. Mean number of *B. dorsalis* (red) and *C. capitata* (green) flies captured in the treatment and control plots of the commercial farms (UAAIE) over the course of 35 weeks. Graphs were made using ggplot2 (Wickham, 2010), URL: <http://ggplot2.tidyverse.org>. using R (version 3.53.12, R Core Team, 2018), URL: <http://www.R-project.org/>.

Reference

R Core Team, R (2018) A language and environment for statistical computing. Vienna, Austria, *R Foundation for Statistical Computing*. URL: <http://www.R-project.org/>.

Wickham H (2010), ggplot2 (Version: 3:0:0): Elegant Graphics for Data Analysis. *J Stat Softw* 35:65-88. URL: <http://ggplot2.tidyverse.org>.
